# Test-retest reliability and minimal detectable change of the Cognitive Abilities Screening Instrument in patients with dementia

**DOI:** 10.1371/journal.pone.0216450

**Published:** 2019-05-07

**Authors:** En-Chi Chiu, Ping-Keung Yip, Peter Woo, Yi-Te Lin

**Affiliations:** 1 Department of Long-Term Care, National Taipei University of Nursing and Health Sciences, Taipei, Taiwan; 2 School of Medicine, College of Medicine, Fu Jen Catholic University, New Taipei City, Taiwan; 3 Department of Neurology, Cardinal Tien Hospital, New Taipei City, Taiwan; 4 Deputy Superintendent, Cardinal Tien Hospital Yung Ho Branch, New Taipei City, Taiwan; Istituto Di Ricerche Farmacologiche Mario Negri, ITALY

## Abstract

**Background:**

The Cognitive Abilities Screening Instrument (CASI) is widely used to assess global cognitive function in patients with dementia. It contains nine cognitive domains, namely long-term memory, short-term memory, attention, mental manipulation, orientation, abstraction and judgment, language, visual construction, and list-generating fluency. However, test-retest reliability and minimal detectable change (MDC) of the CASI are largely unknown in patients with dementia, which limits its utility and the explanation of a score change.

**Purpose:**

The purpose of this study was to examine test-retest reliability and calculate MDC of the CASI in patients with dementia.

**Methods:**

Fifty-two patients with dementia completed the CASI twice with a two-week interval. The frequencies of the scores in the Clinical Dementia Rating (0.5, 1, and ≥ 2) were 38.5, 36.5, and 25.0, respectively. Test-retest reliability was examined using intraclass correlation coefficient (ICC) for the total score and nine domains of the CASI. The MDC was calculated based on standard error of measurement.

**Results:**

The ICC value of the CASI total score was 0.97 while the ICC value for the nine domains were 0.65–0.92. The MDC values (MDC%) were 11.6 (12.9%), 2.8 (23.2%), 4.5 (41.2%), 3.4 (42.1%), 4.9 (49.2%), 5.3 (29.2%), 3.4 (28.8%), 2.2 (22.3%), 3.2 (32.1%), and 3.1 (30.7%) for CASI total score, long-term memory, short-term memory, attention, mental manipulation, orientation, abstraction and judgment, language, visual construction, and list-generating fluency, respectively.

**Conclusion:**

Our results revealed that the CASI has sufficient test-retest reliability. The MDC values are useful in determining a real change (i.e., improvement or deterioration) between two assessments of an individual patient. However, four domains (i.e., short-term memory, attention, mental manipulation, and list-generating fluency) demonstrated lower ICC values and substantial random measurement errors. Clinicians and researchers should be cautious while using these four domains to explain score changes between repeated assessments of patients with dementia.

## Introduction

Along with the trend of global aging, the population suffering from dementia is rapidly increasing. According to a report by the World Health Organization, 7.7 million new cases of dementia are diagnosed every year [1]. The prevalence of dementia is about 8%-14% [2, 3]. Cognitive impairment is one of the main characteristics of patients with dementia and it is important for clinical diagnosis. Common signs of cognitive impairment that appear in patients with dementia include impaired memory, attention, language, and executive function [4]. These cognitive impairments influence patients’ performance of activities of daily living, work, and social interaction, which exerts great burdens on caregivers and influences the quality of life of both patients and caregivers [5, 6]. Therefore, a measure of global cognitive function should be applied to develop treatment plans and conduct outcome studies for patients with dementia in clinical and research settings.

The Cognitive Abilities Screening Instrument (CASI) is widely used to assess global cognitive function in patients with dementia [7, 8]. It was developed based on symptom profiles in the diagnosis of dementia and three cognitive measures (i.e., the Mini Mental State Examination, the Modified Mini-Mental State test, and the Hasegawa Dementia Screening Scale). Thus, the CASI was theoretically framed on nine cognitive domains. The factor structures of the nine cognitive domains of the CASI were supported using confirmatory factor analysis [9]. The CASI has three characteristics. First, it can be applied cross-culturally in measuring the severity of dementia. Second, it contains nine domains that comprehensively measure patients’ cognitive function; it also screens patients’ profiles of cognitive function to determine weaknesses and strengths in multiple dimensions. Third, the MMSE score can be extracted directly from the CASI [7, 10, 11]. Therefore, the CASI is a common measure applied for general use in patients with dementia.

Reliability is the extent to which the results of a measure are consistent and accurate over time [12]. Test-retest reliability can be estimated by the level of consistency between repeated assessments using the intraclass correlation coefficient (ICC) [13]. The ICC is a kind of relative reliability index that can be applied to compare reliability between measures. Minimal detectable change (MDC) is a type of absolute reliability index [14]. It is the minimal threshold of change that falls beyond the random measurement error at a certain confidence level (generally 95%) between two assessments [15]. The MDC value can be used to interpret an individual patient’s score change as a real change (i.e., improvement or deterioration). Thus, to investigate the reliability of a cognitive measure, test-retest reliability and MDC should be evaluated.

The construct validity of the CASI has been examined (e.g., factor structures) [9]. However, its test-retest reliability and MDC have not been examined in patients with dementia, which limits its utility and explanation of score change. Therefore, the main purpose of this study was to examine the test-retest reliability and calculate the MDC of the CASI. We also examined the internal consistency and convergent validity (i.e., correlations among nine domains) of the CASI in patients with dementia.

## Methods

### Participants

This was a prospective study. Patients with dementia were recruited from one hospital, one elder care center, and two day-care centers in northern Taiwan between May and December 2017. They were included if they met the following criteria: (1) diagnosis of dementia based on the Diagnostic and Statistical Manual of Mental Disorders, fifth edition; (2) age ≥ 50 years; and (3) patients in a stable condition with a stable dose of medication within the past month. The criteria for patients excluded from the study were: (1) history of severe brain injury; (2) diagnoses of intellectual disability; and (3) different scores on the Clinical Dementia Rating (CDR) between two assessments. We did not set the recruiting criteria for the cognitive capacity of patients. Patients and their caregivers both needed to sign the informed consent to indicate willingness to participate in this study. This study was approved by the Institutional Review Board of Cardinal Tien Hospital. A sample size of at least 46 participants for reliability was estimated with a power of 0.80 at a significance level of 0.05 [16].

### Procedures

The CASI and the CDR were administered to participants twice in two separate weeks. The CDR was applied to reflect the severity of dementia in this study. The participants were assessed in a quiet space to avoid interferences that could affect their responses. Their demographic data were collected from the medical charts.

### Measures

The CASI measures global cognitive function and contains nine cognitive domains: long-term memory (scores range from 0–10), short-term memory (scores range from 0–12), attention (scores range from 0–8), mental manipulation (scores range from 0–10), orientation (scores range from 0–18), abstraction and judgment (scores range from 0–12), language (scores range from 0–10), visual construction (scores range from 0–10), and list-generating fluency (scores range from 0–10). The long-term memory domain assesses the ability to recall general knowledge (e.g., in what direction does the sun set?). The short-term memory domain assesses the ability to retain information provided in a short time (e.g., recalling objects that were just seen). The attention domain assesses the ability to repeat words and sentences. The mental manipulation domain assesses arithmetic ability (e.g., serial subtractions of 3s). The orientation domain assesses the ability of orienting time, place, and age. The abstraction and judgment domain assesses the ability of problem solving (e.g., what actions would you take if you saw your neighbor’s house catching fire?). The language domain assesses the abilities of reading, writing, naming, and following commands. The visual construction domain assesses the ability to copy two intersecting pentagons. The list-generating fluency domain assesses the ability to list four-legged animals. The CASI total score is the sum of the nine domains’ scores. The range of the total score is 0–100. A higher score indicates better global cognitive function [8].

The CDR assesses cognitive and functional impairments in patients with dementia. It comprises six domains: memory, orientation, judgment and problem solving, community affairs, home and hobbies, and personal care. Except for the personal care domain, all other domains are rated on five levels of impartment (0–0.5-1-2-3). The personal care domain is rated on four levels of impartment (0-1-2-3). A global score is estimated from the six domains, which quantifies the severity of dementia: 0 (healthy), 0.5 (questionable dementia), 1 (mild dementia), 2 (moderate dementia), and 3 (severe dementia) [17]. The CDR has sufficient reliability and validity in patients with dementia [18].

### Data analysis

We calculated ICC_2, 1_ on the basis of a two-way random model with an absolute agreement type. The criteria for the values of ICC were as follows: > 0.80, excellent reliability; 0.61–0.80, good reliability; 0.41–0.60, moderate reliability; and ≤ 0.40, poor reliability [19]. The MDC values of the domains and total CASI score were calculated using the following formula [15]:

$$\mathrm{SEM}={\mathrm{SD}}_{1}\times \sqrt{1-ICC}$$ (1)

$$\mathrm{MDC}=z‑\mathrm{value}\times \mathrm{SEM}\times \sqrt{2}$$ (2)

where SEM is standard error of measurement, SD_1_ is standard deviation in the first assessment, and z-value is the confidence interval (CI) from a normal distribution (e.g., 1.96 for 95% CI).

The percentage of MDC (hereafter MDC%) was computed to present a relative amount of random measurement errors. The MDC% was computed as the MDC divided by the maximum score and multiplied by 100. The criterion for an acceptable random measurement error was MDC% < 30% [20, 21].

We used two methods to evaluate systematic bias: paired *t*-test and effect size. The paired *t*-test (two-tailed, α = 0.05) was to examine whether a statistically significant difference occurred between two assessments, while effect size (Cohen’s *d*) was to estimate the size of systematic bias. The criteria for Cohen’s *d* were: ≥ 0.80, large effect size; 0.50–0.79, moderate effect size; and 0.20–0.49, small effect size [22].

We applied the Bland-Altman plot with 95% limits of agreement (LOA) to visualize the agreement between the two assessments. The Bland-Altman plot plotted the differences of the two assessment scores against the mean scores of the two assessments. The LOA was estimated as the mean difference ± 1.96 × SD of the difference [23]. The plot was used to inspect the data for heterogeneity in the distribution of the values of the differences for the CASI total score and individual domains (heteroscedasticity). Heteroscedasticity was examined using Pearson’s *r* between the mean values and the absolute differences of the two assessments. The data were considered to exhibit heteroscedasticity when *r* > 0.30 [24].

Internal consistency was evaluated using Cronbach’s alpha (α). The standards of α were ≥ 0.70 for group comparisons and ≥ 0.90 for individual comparisons [25]. For examining convergent validity, we calculated the correlations (Pearson’s *r*) among the nine domains of the CASI. The criteria of sufficient convergent validity were: 0.40 ≤ *r* < 0.70 indicating moderate correlation, and *r* > 0.70 indicating strong correlation [26].

## Results

Fifty-two participants finished the assessments twice in two weeks. Their mean age was 80.7 years, 48.1% of the participants were male, and 61.5% of participants had CDR ≥ 1.0. We recruited 10, 17, 13, and 12 participants from one hospital, one elderly care center, one day-care center, and another day-care center, respectively. Table 1 presents the demographics and clinical characteristics of the participants. The data used in this study is provided in the S1 Appendix.

**Table 1:** Demographics and clinical characteristics of participants (n = 52).

Characteristic	
Age (years), mean (SD)	80.7 (8.2)
Gender, n (%)	
Male	25 (48.1)
Female	27 (51.9)
Marital status	
Married	22 (42.3)
Divorced	2 (3.8)
Widowed	28 (53.8)
Education, n (%)	
Elementary school and below	22 (42.3)
Junior high school	7 (13.5)
Senior high school	9 (17.3)
College and above	14 (26.9)
CDR, n (%)	
0.5	20 (38.5)
1.0	19 (36.5)
2.0	9 (17.3)
3.0	4 (7.7)

SD: standard deviation; CDR: Clinical Dementia Rating.

The ICC of the CASI total score was 0.97 (Table 2). In five domains (i.e., long-term memory, orientation, abstraction and judgment, language, and visual construction), the ICC values were between 0.86–0.92, while in the other four domains (i.e., short-term memory, attention, mental manipulation, and list-generating fluency), the ICC values were between 0.65–0.71. The MDC (MDC%) of the total score was 11.6 (12.9%). The MDC values of the nine domains were between 2.2–5.3 (Table 2). The MDC% of the five domains (i.e., short-term memory, attention, mental manipulation, visual construction, and list-generating fluency) were > 30% (30.7%-49.2%), while the MDC% of the other four domains were < 30% (22.3%-29.2%).

**Table 2:** Results of reliability in the CASI.

Total score and domain score	Mean_1_ (SD_1_)	Mean_2_ (SD_2_)	Difference Mean (SD)	ICC (95% CI)	SEM	MDC (MDC%)	Pearson’s *r*	paired *t*-test (*p*-value)	Cohen’s *d*
CASI total score	51.6 (23.8)	51.1 (22.2)	-0.51 (5.81)	0.97 (0.95, 0.98)	4.2	11.6 (12.9%)	0.14	-0.63 (0.531)	0.02
Long-term memory	7.5 (3.1)	7.4 (3.2)	-0.10 (1.46)	0.90 (0.83, 0.94)	1.0	2.8 (23.2%)	0.02	-0.48 (0.637)	0.03
Short-term memory	3.1 (3.0)	3.0 (2.9)	-0.11 (2.25)	0.71 (0.54, 0.82)	1.6	4.5 (41.2%)	0.36	-0.36 (0.723)	0.04
Attention	5.6 (2.1)	6.0 (1.7)	0.46 (1.53)	0.67 (0.49, 0.80)	1.2	3.4 (42.1%)	-0.45	2.18 (0.034)	0.24
Mental manipulation	5.1 (3.2)	3.7 (2.5)	-1.38 (1.94)	0.69 (0.32, 0.85)	1.8	4.9 (49.2%)	0.41	-5.14 (<0.001)	0.49
Orientation	7.3 (5.9)	6.9 (5.6)	-0.35 (2.62)	0.90 (0.83, 0.94)	1.9	5.3 (29.2%)	0.18	-0.95 (0.346)	0.06
Abstraction and judgment	5.7 (3.3)	5.7 (3.1)	0.00 (1.71)	0.86 (0.76, 0.92)	1.2	3.4 (28.8%)	0.13	0.00 (1.000)	0.00
Language	7.4 (2.8)	7.7 (3.0)	0.26 (1.16)	0.92 (0.86, 0.95)	0.8	2.2 (22.3%)	-0.22	1.61 (0.115)	0.09
Visual construction	6.8 (3.7)	7.0 (3.5)	0.25 (1.58)	0.90 (0.84, 0.94)	1.2	3.2 (32.1%)	-0.05	1.14 (0.260)	0.07
List-generating fluency	3.3 (1.9)	3.7 (2.4)	0.50 (1.78)	0.65 (0.46, 0.78)	1.1	3.1 (30.7%)	0.43	2.03 (0.048)	0.23

SD: standard deviation; ICC: intra-class correlation coefficient; CI: confidence interval; SEM: standard error of measurement; MDC: minimal detectable change.

Regarding the systematic bias, the paired *t*-test showed a statistically significant difference between the two assessments (*p* < 0.05) in three domains (i.e., attention, mental manipulation, and list-generating fluency) (Table 2). The effect sizes of these domains were between 0.24–0.49. The paired *t*-test of the total score and the other six domains showed no significant differences (*p* = 0.115–1.000) and the effects sizes were between 0.00–0.09.

**Fig 1:**
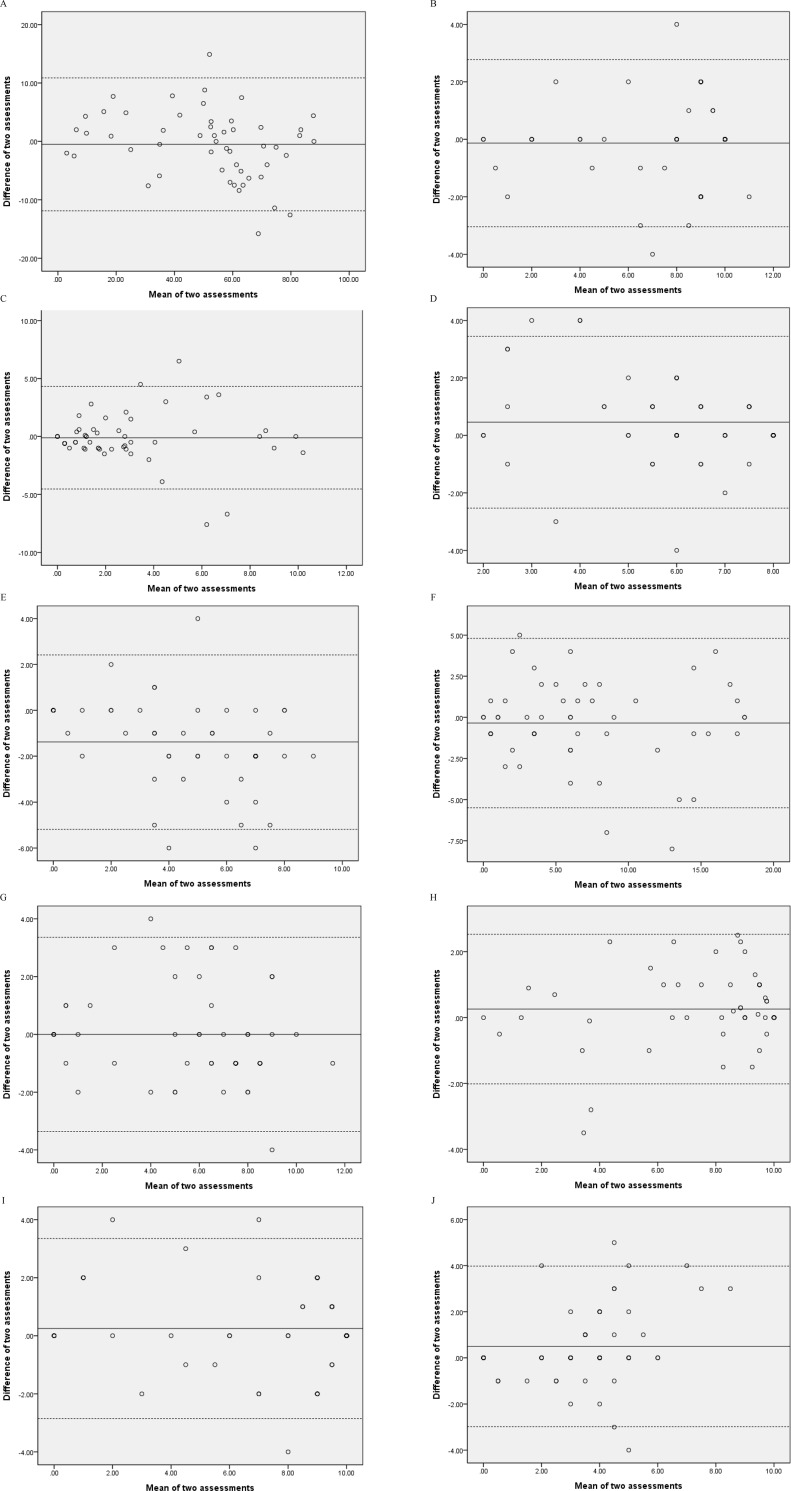
Bland-Altman plots. (A) total score; (B) long-term memory; (C) short-term memory; (D) attention; (E) mental manipulation; (F) orientation; (G) abstraction and judgment; (H) language; (I) visual construction; (J) list-generating fluency. The bold line is the mean difference. The two dotted lines are the 95% limits of agreement.

The Bland-Altman plots of the CASI total score and domains are depicted in Fig 1. The LOAs of the CASI were [-11.9, 10.9] for total score, [-3.0, 2.8] for long-term memory, [-4.5, 4.3] for short-term memory, [-2.5, 3.5] for attention, [-5.2, 2.4] for mental manipulation, [-5.5, 4.8] for orientation, [-3.4, 3.4] for abstraction and judgment, [-2.0, 2.5] for language, [-2.9, 3.4] for visual construction, and [-3.0, 4.0] for list-generating fluency. Correlations between the mean and the difference of the two assessments for the total score and domains were *r* ≤ 0.30, except for four domains (i.e., short-term memory, attention, mental manipulation, and list-generating fluency) (*r* = 0.36–0.45) (Table 2).

Cronbach’s α of the CASI was 0.92. The correlations among the nine domains were *r* = 0.41–0.85 (Table 3).

**Table 3:** Correlations (*r*) among nine domains of the CASI.

Domain	Long-term memory	Short-term memory	Attention	Mental manipulation	Orientation	Abstraction and judgment	Language	Visual construction
Short-term memory	0.50							
Attention	0.69	0.49						
Mental manipulation	0.69	0.41	0.55					
Orientation	0.60	0.72	0.42	0.56				
Abstraction and judgment	0.81	0.58	0.72	0.73	0.67			
Language	0.85	0.51	0.76	0.73	0.61	0.83		
Visual construction	0.70	0.44	0.60	0.60	0.57	0.70	0.78	
List-generating fluency	0.75	0.55	0.60	0.48	0.57	0.72	0.67	0.67

## Discussion

According to ICC values, the CASI total score showed excellent test-retest reliability, and the domains revealed good to excellent test-retest reliability. Four domains (i.e., short-term memory, attention, mental manipulation, and list-generating fluency) represent lower ICC values. The MDC% of these four domains were > 30%, indicating large random measurement error. The reasons for relative insufficient reliability of these domains may be patients’ symptoms. In our study, 38.5% (n = 20) of participants had questionable dementia. In the early stage of dementia, patients have difficulties with regard to short-term memory, concentration, and verbal fluency [27, 28]. They may manifest unstable cognitive functions, which increase the instability of responding to related cognitive items in the short-term memory, attention, mental manipulation, and list-generating fluency domains. The ceiling effect may be the reason the MDC% of the visual construction domain was > 30%. In the first and second assessments, 34.6% and 38.5% of patients obtained the highest score in the visual construction domain, respectively. The ceiling effect indicates that the visual construction domain could not distinguish patients with high ability in visual construction skills. To reduce high MDC% of the visual construction domain, copying more difficult figures could be considered. As a whole, the CASI is a reliable measure for following up changes to global cognitive function in patients with dementia.

Our results showed no statistically significant difference of the paired *t*-tests and negligible effect sizes in the CASI total score and six domains, thereby demonstrating no systematic biases. For the other three domains (i.e., attention, mental manipulation, and list-generating fluency), systematic biases were noticed with significant difference of the paired *t*-tests and small effect sizes. In the Bland-Altman plots, the mental manipulation domain revealed that the mean difference were not close to zero, indicating obvious systematic bias. Four domains (i.e., short-term memory, attention, mental manipulation, and list-generating fluency) revealed heteroscedasticity, representing a trend: differences between repeated assessments decrease/increase as the mean values of the assessment decrease/increase [29]. A possible reason for systematic bias or heteroscedasticity is item difficulty. In the first assessment, 9.6% and 13.5% of patients obtained the lowest score (0) in the short-term memory and mental manipulation domains, respectively. In the second assessment, a higher percentage (13.5%-17.3%) of patients obtained the lowest score in these two domains. Patients may ask why the same assessments needed to be administered again; they may feel like they were taking exams. Their emotions may influence their cognitive performance in the second assessment [30]. Further, users may consider not conducting the CASI within a short period (e.g., a two-week interval), which may influence patients’ willingness to undergo the assessment.

The Cronbach’s α of the CASI was > 0.90, indicating the CASI total score can be applied in group comparison studies and monitoring individual scores. Moderate to strong correlations were found among the nine domains of the CASI, demonstrating the nine domains may measure a similar construct (e.g., global cognitive function). According to our results, the CASI reveals good internal consistency and convergent validity in patients with dementia.

Regarding its clinical implications, the MDC values of the CASI total score and domains were provided in this study. A score change for an individual patient greater than the MDC value can be viewed as a real change with 95% certainty. Clinicians and researchers can therefore use the MDC value to explain patients’ scores accurately. The other implication is the determination of the treatment effect by using the MDC value as a threshold to calculate the MDC proportion [15]. The MDC proportion is the proportion of participants’ score change greater than the MDC value. For instance, a sample size of 50 revealed that 10 participants had a score change > 11.6 in the CASI total score, which showed that the MDC proportion was 20% in an intervention for improving performance in global cognitive function. Researchers can compare the MDC proportions between experimental and control groups. The group with a higher MDC proportion exhibits a greater treatment effect. Besides reporting a statistically significant change, the MDC proportion can be addressed in a clinical trial study.

Two limitations were noticed in this study. First, participants were recruited only from northern Taiwan and the sample size was small, which limits the generalizability of our results. The period of repeated assessment was short (2 weeks), which may not provide much insight into the cognitive status of the sample with chronic nature. Future studies could compare our CASI findings with larger sample sizes and longer periods of repeated assessments. Second, we did not examine the concurrent validity using other global cognitive measures (e.g., the Mini Mental State Examination and the Montreal Cognitive Assessment), neither did we evaluate the diagnostic ability (sensitivity and specificity) and nor test the ability to detect change (e.g., minimal clinical important difference). Future studies are warranted to examine the concurrent validity, sensitivity, specificity, minimal clinical important difference (by caregiver or physician) of the CASI in patients with dementia.

## Conclusion

Our results revealed that the CASI has sufficient test-retest reliability. However, four domains (i.e., short-term memory, attention, mental manipulation, and list-generating fluency) demonstrated lower ICC values and substantial random measurement errors. Clinicians and researchers should be cautious while explaining the score changes in these four domains between repeated assessments for patients of dementia.

## Supporting information

S1 Appendix Raw data of the study. (XLSX)
